# The protective effect of apolipoprotein H in paediatric sepsis

**DOI:** 10.1186/s13054-024-04809-2

**Published:** 2024-01-30

**Authors:** Zhicai Yu, Changxue Xiao, Rong Liu, Dandan Pi, Bian Jin, Zhen Zou, Feng Xu

**Affiliations:** 1 Department of Critical Care Medicine, Children’s Hospital of Chongqing Medical University, National Clinical Research Center for Child Health and Disorders, Ministry of Education Key Laboratory of Child Development and Disorders, Chongqing Key Laboratory of Pediatric Metabolism and Inflammatory Diseases, Chongqing, China; 2 Molecular Biology Laboratory of Respiratory Disease, Institute of Life Sciences, Chongqing Medical University, Chongqing, 400016 China; 3 Department of Pediatric Intensive Care Unit, Children’s Hospital Affiliated to Chongqing Medical University, Chongqing, 400014 China

**Keywords:** Sepsis, Apolipoprotein H, Macrophage polarization, TLR4/NF-κB

## Abstract

**Background:**

Sepsis is a severe condition characterized by acute organ dysfunction resulting from an imbalanced host immune response to infections. Apolipoprotein H (APOH) is a critical plasma protein that plays a crucial role in regulating various biological processes. However, the precise role of APOH in the immunopathology of paediatric sepsis remains unclear.

**Methods:**

In this study, we evaluated the concentration of APOH in paediatric patients with sepsis and healthy individuals. In an experimental sepsis model of caecal ligation and puncture (CLP), the impact of APOH on survival, organ injury, and inflammation was measured. Furthermore, the anti-inflammatory effects of APOH were investigated across diverse immune cell types, encompassing peripheral blood mononuclear cells (PBMCs), peritoneal macrophages (PMs), bone marrow-derived macrophages (BMDMs), and RAW 264.7 macrophages.

**Results:**

In the pilot cohort, the relative abundance of APOH was found to be decreased in patients with sepsis (2.94 ± 0.61) compared to healthy controls (1.13 ± 0.84) (*p* < 0.001), non-survivors had lower levels of APOH (0.50 ± 0.37) compared to survivors (1.45 ± 0.83) (*p* < 0.05). In the validation cohort, the serum concentration of APOH was significantly decreased in patients with sepsis (202.0 ± 22.5 ng/ml) compared to healthy controls (409.5 ± 182.9 ng/ml) (*p* < 0.0001). The application of recombinant APOH protein as a therapeutic intervention significantly lowered the mortality rate, mitigated organ injury, and suppressed inflammation in mice with severe sepsis. In contrast, neutralizing APOH with an anti-APOH monoclonal antibody increased the mortality rate, exacerbated organ injury, and intensified inflammation in mice with non-severe sepsis. Intriguingly, APOH exhibited minimal effects on the bacterial burden, neutrophil, and macrophage counts in the sepsis mouse model, along with negligible effects on bacterial phagocytosis and killing during *Pseudomonas aeruginosa* infection in PMs, RAW 264.7 cells, and PBMCs. Mechanistic investigations in PMs and RAW 264.7 cells revealed that APOH inhibited M1 polarization in macrophages by suppressing toll-like receptor 4 (TLR4)/nuclear factor-κB (NF-κB) signalling pathway.

**Conclusion:**

This proof-of-concept study demonstrated that APOH has a protective role in the host defense response to sepsis, highlighting the potential therapeutic value of APOH in sepsis treatment.

**Graphical abstract:**

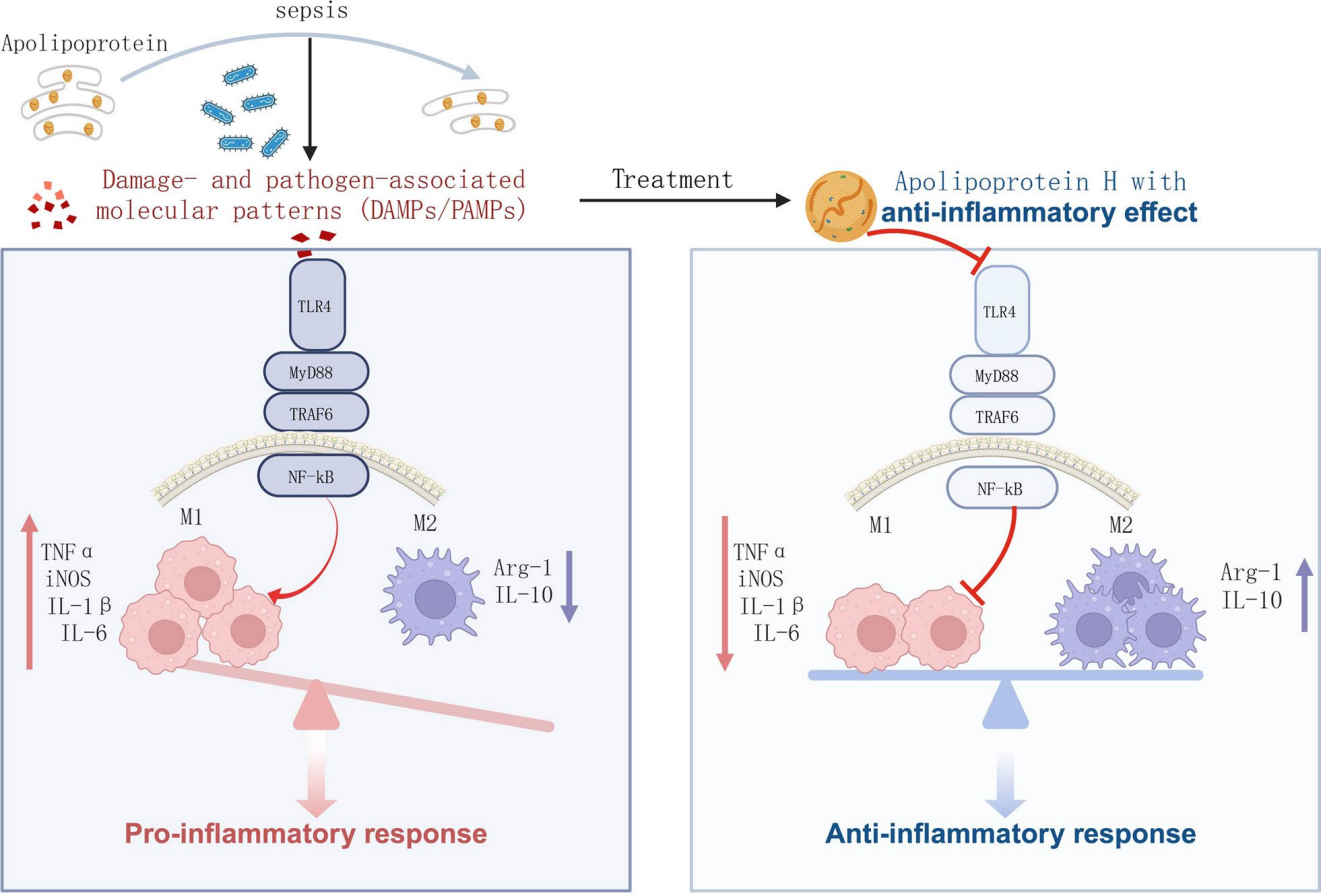

**Supplementary Information:**

The online version contains supplementary material available at 10.1186/s13054-024-04809-2.

## Introduction

Sepsis, a condition characterized by the dysregulation of the body's response to infection, leading to organ injury, stands as the main cause of mortality in children [1–3]. Approximately 40% of sepsis cases occur in children under the age of 5, and it is alarming that a staggering 20 million cases were reported worldwide in 2017 alone [4]. Despite the numerous clinical trials conducted, the quest for effective drugs to combat sepsis remains an immensely challenging undertaking.

Apolipoprotein H (APOH), also known as beta-2-glycoprotein I (β2-GPI), is a highly abundant plasma protein with a molecular size of approximately 50 kDa. It is primarily synthesized by liver cells and comprises five domains (I–V) [5]. APOH is a complement regulator across diverse biological processes and conditions, including antiphospholipid syndrome (APS), the procoagulant state, diabetes, and immune disorders [6–8]. Furthermore, studies have indicated APOH’s involvement in innate immunity by binding to viruses and bacteria [9, 10]. Despite the compelling evidence supporting the role of APOH in numerous diseases, a clear correlation between the presence of APOH and the onset/progression of paediatric sepsis remains elusive.

In delving into the potential role of APOH in sepsis, we assessed the circulating levels of APOH in paediatric patients with sepsis in both the pilot and validation cohorts. Furthermore, we utilized a clinically relevant model of sepsis induced by caecal ligation and puncture (CLP), along with multiple types of macrophages (including peritoneal macrophages (PMs), bone marrow-derived macrophages (BMDMs), peripheral blood mononuclear cells (PBMCs), and RAW 264.7 cells), to explore the involvement of APOH in host defense and unravel the underlying molecular mechanism.

## Materials and methods

### Detailed methods are available in the online supplement (Additional file 1)

#### Study population

In the pilot cohort, a total of sixty-eight paediatric patients were recruited from the Paediatric Intensive Care Unit (PICU) of the Children’s Hospital of Chongqing Medical University between December 2016 and October 2018. These patients met the clinical criteria for the International Paediatric Sepsis Conference 2005 [11]. Among the recruited patients, there were 42 survivors and 26 non-survivors. Additionally, twenty-six healthy volunteers were included as controls. In this cohort, peripheral blood samples were collected from each group and pooled into six samples for analysis using liquid chromatography tandem mass spectrometry (LC–MS/MS).

In the validation cohort, thirty-six paediatric patients with sepsis were enrolled at admission to the PICU of Children’s Hospital of Chongqing Medical University between January 2022 and March 2023. Peripheral blood was collected at 24 h, 48 h, and 72 h after admission. The collected serum was isolated and stored at -80 °C until further analysis. Various clinical data were recorded at 24 h after admission, including sex, age, white blood cells (WBCs), procalcitonin (PCT), C-reactive protein (CRP), Paediatric Sequential Organ Failure (pSOFA), Paediatric Critical Illness Score (PCIS), and systemic inflammatory response syndrome (SIRS) score. Additionally, information on microbiological findings, ventilation, use of adrenocortical hormone, renal replacement therapy, length of PICU stays, and mortality during the 28-day study period was recorded. Sixty-nine healthy control blood samples were taken from healthy donors.

The protocols described in this study were approved by the Clinical Research Ethics Committee of the Institutional Review Board of Children’s Hospital of Chongqing Medical University (File No: ChiCTR-ROC-17011164 and (2022) Ethical Review Research No. 427). Informed consent was obtained from all participants in accordance with the principles outlined in the Declaration of Helsinki.

#### Experimental animals

All animal experiments were conducted following the guidelines provided by the Chongqing Experimental Animal Center and the Animal Committee of Children’s Hospital (CHCMU-IACUC20221227002). C57BL/6 mice were obtained from Chongqing Medical University and bred in a controlled environment with a temperature range of 20–24 °C and a 12-h light/dark cycle. They were provided with free access to standard chow and water. To establish a model of polymicrobial sepsis, caecal ligation and puncture (CLP) was performed [12]. Prior to the surgery, the mice were anesthetized intraperitoneally with pentobarbital sodium (75 mg/kg body weight). A 1 cm incision was made in the midline abdomen after disinfecting the skin, and the caecum was exposed. The caecum was ligatured at either 60% (severe CLP, 20–40% survival) or 40% (non-severe CLP, 60–80% survival) and punctured with a 21-gauge needle. Then, the caecum was placed back into the abdominal cavity and the incisions were closed. Intraperitoneal injection of normal saline (0.05 ml/g body weight) was administered for resuscitation, and water and lab chow were reintroduced after the operation. Survival of the mice was monitored daily from Day 2 to Day 14 after the surgery. A humane endpoint was used for the CLP-treated mice.

#### In vivo administration of recombinant APOH

The mice were divided into two groups: CLP + 0.1% bovine serum albumin (BSA) and CLP + recombinant murine apolipoprotein H (rAPOH). In the rAPOH group, mice were intraperitoneally injected with 20 μg of murine rAPOH (R&D Systems, 6575-AH-050, USA) dissolved in 100 μl of phosphate buffer solution (PBS). In the BSA group, mice received 0.1% BSA in 100 μl of PBS instead.

#### Antibody-mediated blockade of APOH

To neutralize APOH activity in non-severe CLP, mice in the CLP + anti-APOH (anti-APOH) group were injected with 10 μg of anti-mouse APOH neutralizing monoclonal antibody (Proteintech, Monoclonal Mouse IgG1, 66,074-1-Ig, China) dissolved in 100 μl of PBS. Another group of mice was injected with mouse IgG1 antibody as a control (IgG control).

#### In vitro administration of recombinant APOH

RAW 264.7 macrophages were obtained from the Institute of Children's Hospital Affiliated to Chongqing Medical University. PMs, BMDMs, PBMCs, and RAW 264.7 macrophages were cultured in DMEM or 1640 medium supplemented with 10% fetal bovine serum (FBS) and 1% penicillin–streptomycin. The cells were incubated in a 5% CO_2_ incubator at 37 °C. Prior to treatment, the cells were preincubated with recombinant APOH (rAPOH) at a concentration of 20 ng/ml for 30 min. Subsequently, the cells were treated with purified lipopolysaccharide (LPS) (100 ng/ml) obtained from *Escherichia coli* O55:B5 (Abmole, M9524, USA) for 6 h.

### Statistical analysis

Data were presented as the mean ± standard deviation (S.D.). Group differences were determined using a *t* test (Mann‒Whitney U test) or one-way ANOVA (Tukey’s multiple comparisons test). Survival studies were analyzed using log-rank (Mantel‒Cox) tests, and correlations were analyzed using the nonparametric Spearman's rank correlation coefficient. All statistical tests were performed using GraphPad Prism version 9 (GraphPad Software, San Diego, CA). Statistical significance was defined as *P* < 0.05.

## Results

### The concentration of APOH is significantly decreased in paediatric patients with sepsis

**Fig. 1:**
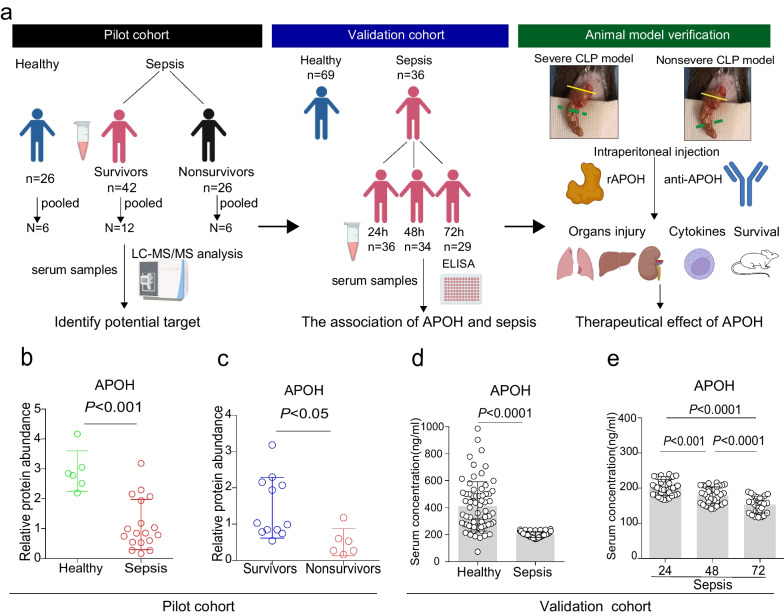
APOH levels are significantly decreased in patients with sepsis. **a** LC–MS/MS was employed to identify potential biomarkers for sepsis in a pilot cohort, and ELISAs were used to confirm the expression of APOH in a validation cohort. A murine model of CLP-induced sepsis was employed to evaluate the therapeutic impact of APOH. **b** LC‒MS/MS was employed to identify the level of APOH in patients with sepsis and healthy controls in the pilot cohort. **c** LC‒MS/MS was employed to identify the level of APOH in survivors and non-survivors with sepsis in the pilot cohort. **d** Serum levels of APOH in 36 paediatric patients with sepsis and 69 healthy controls in the validation cohort. **e** The dynamics of APOH levels in the serum of paediatric patients with sepsis at 24, 48 and 72 h in the validation cohort. The data are presented as the means ± standard deviations (S.D.). “*” indicates a difference between groups. *p < 0.05, **p < 0.01, *** p < 0.001, ****p < 0.0001. Apolipoprotein H, APOH; liquid chromatography tandem mass spectrometry, LC‒MS/MS; enzyme-linked immunosorbent assay, ELISA.

In this study, LC–MS/MS was employed to identify potential biomarkers for sepsis in a pilot cohort, and ELISAs were used to confirm the expression of APOH in a validation cohort. To evaluate the therapeutic impact of APOH, a murine model of CLP-induced sepsis was employed (Fig. 1a). The characteristics of the patients with sepsis and healthy controls in the pilot cohort are presented in Additional file 2: Table S1. The analysis revealed a lower APOH level in sepsis patients compared to healthy controls in pooled samples (Fig. 1b). Moreover, non-survivors with sepsis exhibited decreased expression of APOH compared to survivors in the pilot cohort (Fig. 1c). The expression levels of APOH were subsequently validated in an independent patient cohort, comprising thirty-six sepsis patients and sixty-nine healthy controls. The characteristics of these individuals are outlined in Table 1. Consistent with expectations, serum concentrations of APOH were significantly reduced in patients with sepsis compared to healthy control subjects (Fig. 1d). Furthermore, the levels of APOH showed a progressive decrease as sepsis advanced (Fig. 1e). However, in the validation cohort, no significant difference in APOH was observed between survivors (n = 30, 202.7 ± 23.4 ng/ml) and non-survivors (n = 6, 199.1 ± 18.2 ng/ml).

**Table 1:** Characteristics of paediatric patients with sepsis and healthy controls

Characteristics	Sepsis patients (n = 36)	healthy controls (n = 69)	*P* value
Sex (male/female)	16/20	41/28	0.144
Age (years)	2.54(0.88–6.86)	6.58(4.88–8.09)	0.004
WBC (10^9^/L)	7.94(4.21–13.24)	NA	
CRP (mg/L)	65.23(16.57–99.96)	NA	
Procalcitonin (ng/ml)	2.2(0.40–9.080)	NA	
Infection site (Number of patients)			
Respiratory	31	NA	
Abdominal	4	NA	
Vascular	0	NA	
Urinary	1	NA	
Bacteraemia	1	NA	
Isolates (Number of patients)			
Gram positive	0	NA	
Gram negative	9	NA	
Fungus	0	NA	
Virus	4	NA	
Miscellaneous	13	NA	
Other	10	NA	
pSOFA score	4 (4–7)	NA	
PCIS score	79(74–86)	NA	
SIRS score	3(2–4)	NA	
PICU stay(days)	7 (4–10.75)	NA	
Ventilation	24/36	NA	
Use of adrenocortical hormone	13/36	NA	
Renal replacement therapy	10/36	NA	
Died/survived	6/36	NA	

The data are expressed as the median (interquartile range) unless otherwise indicated. *WBC* white blood cell, *CRP* C-reactive protein, *PCT* procalcitonin, *SOFA* sequential organ failure assessment, *PICU* paediatric intensive care unit, *NA* not applicable

### Administration of recombinant APOH protein protects septic mice

**Fig. 2:**
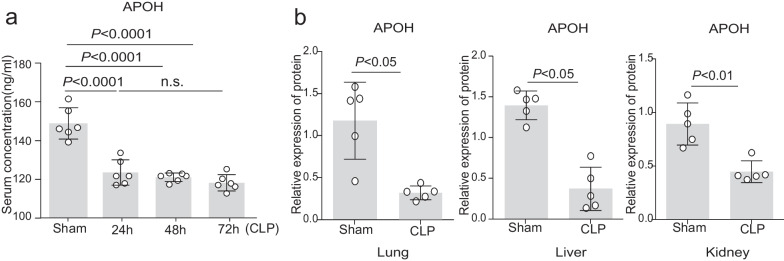
The expression of APOH in mice with CLP. **a** Serum levels of APOH in sham mice and mice with CLP at 24, 48, and 72 h after CLP (n = 6). **b** The relative protein levels of APOH in the lungs, livers, and kidneys (n = 5). The data are presented as the means ± standard deviations (S.D.). “*” indicates a difference between groups. *p < 0.05, **p < 0.01, ***p < 0.001, ****p < 0.0001. Apolipoprotein H, APOH; caecum ligation and puncture, CLP

**Fig. 3:**
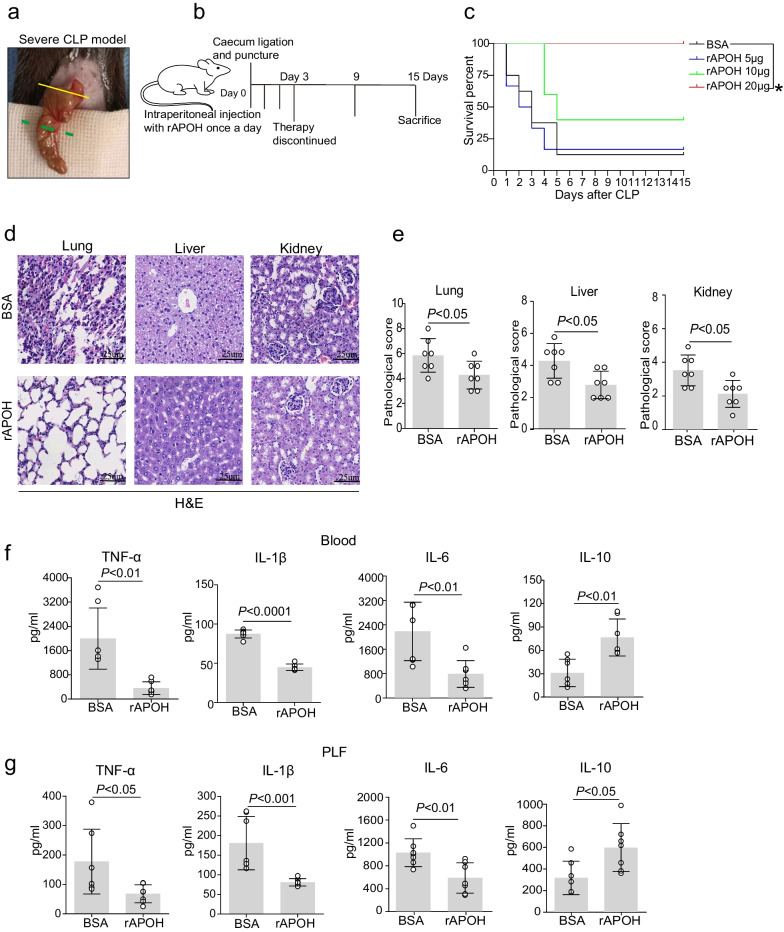
Administration of recombinant APOH protein protects septic mice. **a** Ligation of the caecum at designated positions to establish a severe CLP model. The caecum was ligated (indicated by the dotted green line) at a 60% distance between the distal pole and the base of the caecum (yellow dotted line). **b** The procedures for rAPOH treatment in CLP-induced severe sepsis. **c** Survival of mice with CLP after treatment with BSA and rAPOH (n = 8). **d** Lung, liver, and kidney tissues were stained with H&E in the BSA and rAPOH groups. **e** Semiquantitative scores of tissues were calculated in the BSA and rAPOH groups (n = 7). (**f**) Serum cytokine levels in the BSA and rAPOH groups at 24 h after severe CLP (n = 7). **g** Cytokine levels in the PLF in the BSA and rAPOH groups at 24 h after severe CLP (n = 7). The data are presented as the means ± standard deviations (S.D.). “*” indicates a difference between groups. *p < 0.05, **p < 0.01, ***p < 0.001, ****p < 0.0001. Apolipoprotein H, APOH; recombinant murine APOH, rAPOH; bovine serum albumin, BSA; caecal ligation and puncture, CLP; peritoneal lavage fluid, PLF; haematoxylin and eosin staining, H&E

To illustrate the variation of APOH expression throughout sepsis progression, we determined the serum levels of APOH in polymicrobial sepsis induced by CLP. The concentration of APOH was significantly decreased in CLP-induced mice compared to sham mice throughout the subsequent progression of sepsis (Fig. 2a). Notably, CLP-induced septic mice displayed higher IL-6 levels compared to sham mice (Additional file 3: Fig. S1a). IL-6 levels had a significant negative correlation with APOH expression (Additional file 3: Fig. S1b). In addition, we found that the APOH levels in the lung, liver, and kidney were decreased in the CLP-induced septic mice compared to the sham mice (Additional file 3: Fig. S1c and Fig. 2b). For the induction of severe sepsis resulting in survival rates of 20–40%, the caecum was ligated (indicated by the dotted green line) at a 60% distance between the distal pole and the base of the caecum (yellow dotted line) (Fig. 3a). To further confirm the protective role of APOH in CLP-induced sepsis, we assessed the effects of different doses (5–20 μg) of recombinant murine APOH on severe sepsis in the CLP model (Fig. 3b). The results showed that supplementation with rAPOH (20 μg) significantly improved mouse survival compared to BSA treatment (Fig. 3c). The increased survival of the rAPOH group may be attributed to decreased tissue inflammation and organ injury, as evidenced by amelioration observed in the lung, liver, and kidney (Fig. 3d–e). Moreover, significantly reduced inflammatory cytokines, including TNF-α, IL-1β, and IL-6, while increased IL-10 levels were observed in serum and PLF samples from rAPOH-treated septic mice compared to the BSA control group (Fig. 3f–g).

### Anti-APOH antibody worsens non-severe sepsis in mice

**Fig. 4:**
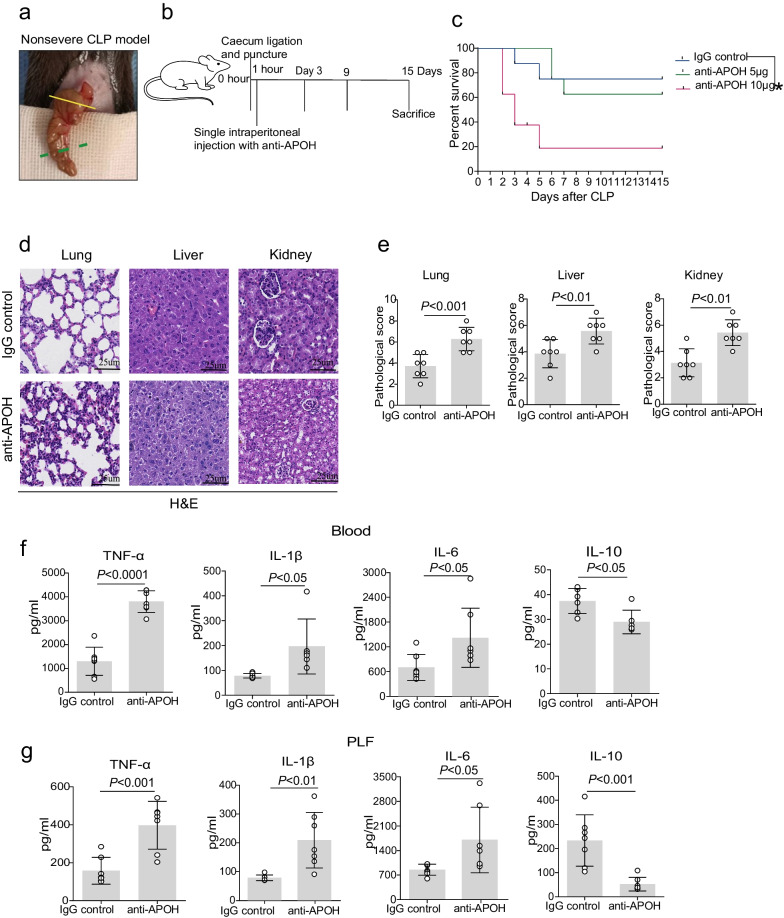
Anti-APOH antibody worsens non-severe sepsis in mice. **a** Ligation of the caecum at designated positions to establish a non-severe CLP model. The caecum was ligated (indicated by the dotted green line) at a 40% distance between the distal pole and the base of the caecum (yellow dotted line). **b** The procedures for the administration of an anti-APOH antibody in mice with CLP-induced non-severe sepsis. **c** Survival of mice with CLP-induced non-severe sepsis after treatment with the anti-APOH antibody (n = 8). **d** Lung, liver, and kidney tissues were stained with H&E in the IgG control and anti-APOH groups. **e** Semiquantitative scores of tissues were calculated in the IgG control and anti-APOH groups (n = 7). **f** Serum cytokine levels in blood in the IgG control and anti-APOH groups at 24 h after non-severe CLP (n = 7). **g** Cytokine levels in the PLF in the IgG control and anti-APOH groups at 24 h after non-severe CLP (n = 7). The data are presented as the means ± standard deviations (S.D.). “*” indicates a difference between groups. *p < 0.05, **p < 0.01, *** p < 0.001, **** p < 0.0001. Apolipoprotein H, APOH; caecal ligation and puncture, CLP; peritoneal lavage fluid, PLF; haematoxylin and eosin staining, H&E

In addition, a non-severe CLP model was established by ligating the caecum (indicated by the dotted green line) at a 40% distance between the distal pole and the base of the caecum (yellow dotted line) (Fig. 4a). To explore the potentially detrimental effects of APOH blockade, we conducted an in vivo study using 5 or 10 μg of anti-APOH antibody (Fig. 4b). As shown in Fig. 4c, the survival rate of septic mice in the anti-APOH group was significantly lower compared to the septic mice treated with the IgG control (Fig. 4c). Furthermore, the septic mice treated with the anti-APOH antibody after CLP exhibited significantly aggravated lung, liver, and kidney injuries compared to the IgG-control mice (Fig. 4d–e). Additionally, the blockade of APOH after CLP led to a significant increase in the levels of serum and peritoneal inflammatory cytokines, such as TNF-α, IL-1β, and IL-6, alongside a decrease in IL-10 levels after non-severe sepsis (Fig. 4f–g). These findings indicate that blocking APOH could exacerbate the severity of sepsis.

### APOH has minimal influence on bacterial phagocytosis and killing capacities upon *Pseudomonas aeruginosa* infection

**Fig. 5:**
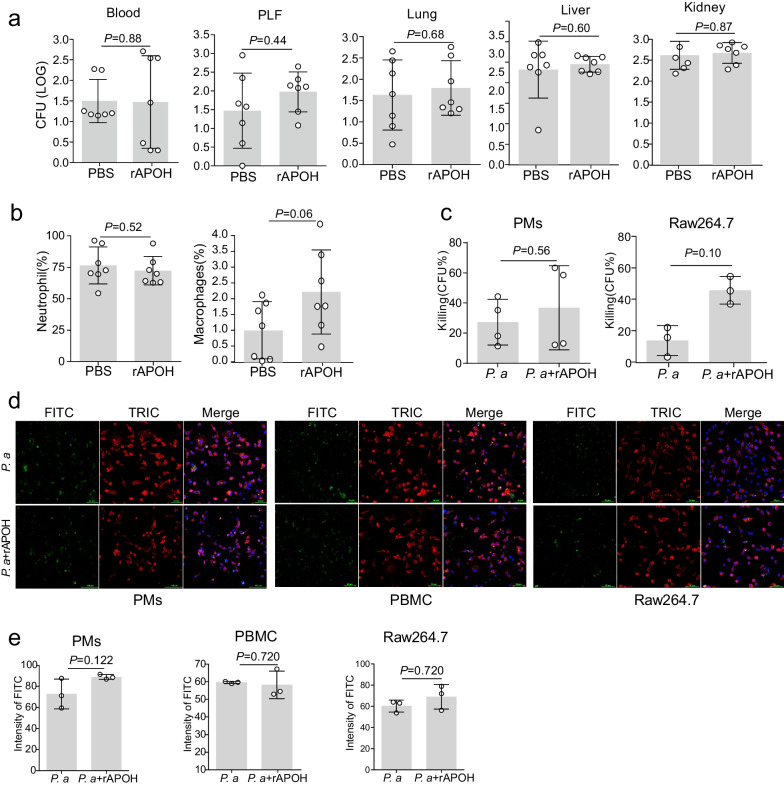
APOH has minimal influence on bacterial phagocytosis and killing capacities upon *Pseudomonas aeruginosa* infection. **a** CFU levels in septic mice treated with rAPOH compared with mice treated with BSA after CLP (n = 7). **b** The proportions of different cell types were observed in the BSA and rAPOH groups (n = 7). **c** Killing rates were determined in the *P.a* and *P.a* + rAPOH groups. **d** Immunofluorescence was employed to detect the effect of APOH on macrophage phagocytosis. **e** Relative fluorescence intensity of FITC-labelled *P.a* in the *P.a.* and *P.a* + rAPOH groups. The data are presented as the means ± standard deviations (S.D.). “*” indicates a difference between groups. *p < 0.05, **p < 0.01. Apolipoprotein H, APOH; recombinant APOH, rAPOH; Bacterial colony-forming unit, CFU; caecal ligation and puncture, CLP; fluorescein isothiocyanate, FITC; peritoneal macrophages, PMs; peritoneal lavage fluids, PLFs; peripheral blood mononuclear cells, PBMCs; *Pseudomonas aeruginosa*, *P.a.*; tetramethylrhodamine-5-isothiocyanate, TRIC

The decreased inflammation levels observed in rAPOH mice can not be attributed to enhanced bacterial clearance. This is because there was no significant difference in local or systemic bacterial colony-forming unit (CFU) levels between the septic mice treated with recombinant APOH and those treated with the BSA (Fig. 5a). Additionally, flow cytometry experiments were conducted to determine the proportions of different cell types of infiltrating leukocytes within the PLF between the rAPOH and BSA groups. The results revealed minimal differences in the proportions of neutrophils and macrophages between the two groups. However, an increasing trend in macrophages was observed after APOH intervention (Additional file 4: Fig. S2a and Fig. 5b). To investigate further the effects of APOH on the intrinsic antibacterial functions of macrophages, PMs and RAW 264.7 macrophages were preincubated with recombinant APOH for 20 h before exposure to *P. aeruginosa*. However, preincubation with APOH did not directly affect the killing capacities (Additional file 4: Fig. S2b and Fig. 5c) or bacterial uptake (Fig. 5d–e) in macrophages upon *P. aeruginosa* infection.

### APOH decreases inflammatory cytokine responses in macrophage cells

**Fig. 6:**
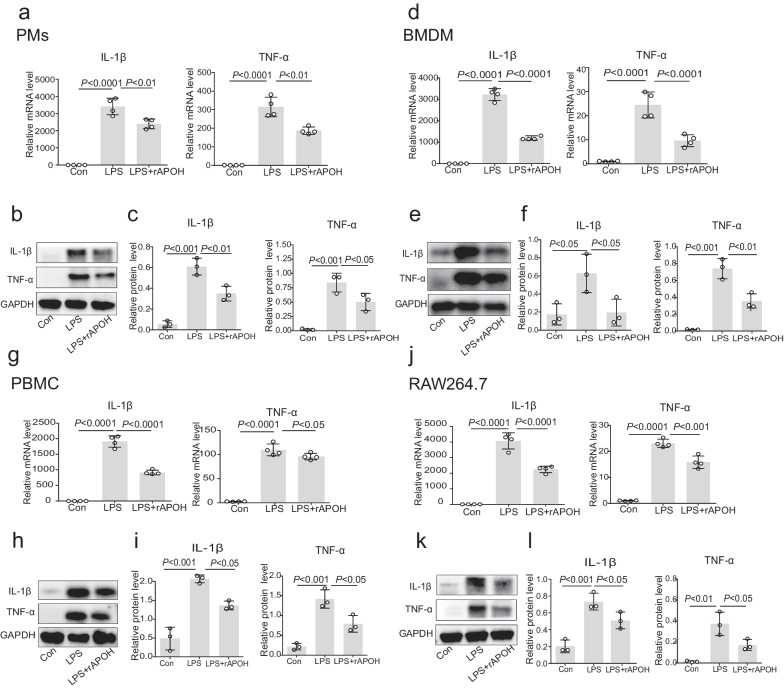
APOH decreases inflammatory cytokine responses in LPS-treated macrophage cells. **a** The mRNA expression levels of IL-1β and TNF-α in PMs were determined by qPCR. **b**–**c** Western blot analysis of the relative expression levels of IL-1β and TNF-α in PMs. **d** The mRNA expression levels of IL-1β and TNF-α in BMDMs were determined by qPCR. **e**–**f** Western blot analysis of the relative expression levels of IL-1β and TNF-α in BMDMs. **g** The mRNA expression levels of IL-1β and TNF-α in PBMCs were determined by qPCR. **h**–**i** Western blot analysis of the relative expression levels of IL-1β and TNF-α in PBMCs. **j** The mRNA expression levels of IL-1β and TNF-α in RAW 264.7 cells were determined by qPCR. **k**–**l** Western blot analysis of the relative expression levels of IL-1β and TNF-α in RAW 264.7 cells. The data are presented as the means ± standard deviations (S.D.). “*” indicates a difference between groups. *p < 0.05, **p < 0.01, *** *p* < 0.001, **** *p* < 0.0001. Apolipoprotein H, APOH; peritoneal macrophages, PMs; lipopolysaccharide, LPS; bone marrow-derived macrophages, BMDMs; peripheral blood mononuclear cell, PBMCs; interleukin, IL; tumour necrosis factor, TNF; Quantitative Real-time reverse transcriptase-polymerase chain reaction, qPCR

**Fig. 7:**
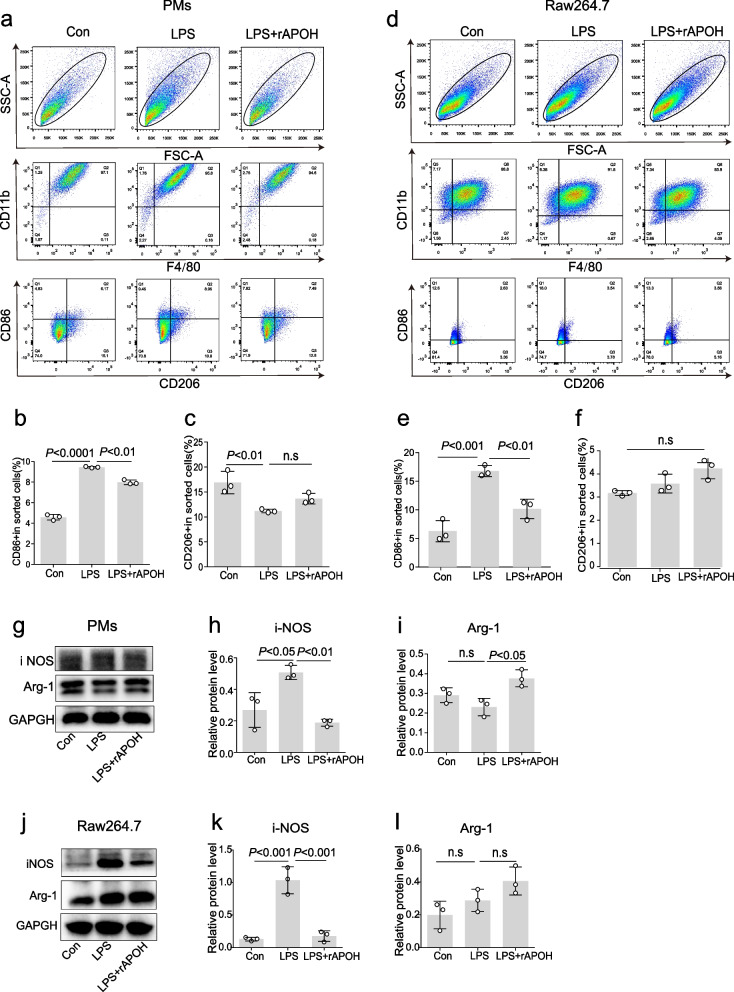
The effect of APOH administration on macrophage polarization. **a** Flow cytometry was employed to determine the change in the M1 and M2 polarization in PMs. **b**–**c** The percentage of M1- and M2-polarized PMs was detected. **d** Flow cytometry was employed to determine the change in the M1 and M2 polarization in RAW 264.7 macrophages. **e**–**f** The percentage of M1- and M2-polarized RAW 264.7 cells were detected. **g** Western blot analysis of iNOS and Arg-1 in PMs. **h**–**i** Relative expression levels of iNOS and Arg-1 in PMs. **j** Western blot analysis of iNOS and Arg-1 in RAW 264.7 cells. **k**–**l** Relative expression levels of iNOS and Arg-1 in RAW 264.7 cells. The data are presented as the means ± standard deviations (S.D.). “*” indicates a difference between groups. *p < 0.05, **p < 0.01, *** *p* < 0.001, **** *p* < 0.0001. Apolipoprotein H, APOH; peritoneal macrophages, PMs; lipopolysaccharide, LPS; inducible nitric oxide synthase, iNOS; Arginase 1, Arg-1

To illustrate the mechanisms contributing to the lower levels of inflammation in rAPOH-treated mice compared to BSA-treated mice, we examined the effects of APOH on cytokine expression in cells challenged with LPS. As depicted in Fig. 6, preincubation with recombinant APOH significantly reduced both the mRNA and protein levels of TNF-α and IL-1β in PMs, BMDMs, PBMCs, and RAW 264.7 cells when contrasted with the LPS-alone group (Fig. 6a–l). This inhibitory effect of APOH on inflammatory cytokines may be associated with the modulation of macrophage polarization from M1 to M2 subtypes. To explore this hypothesis, we assessed the ability of APOH to regulate macrophage polarization in PMs and RAW 264.7 cells. Although no differences were observed in the proportion of CD206 cells and the protein level of Arg-1, preincubation with recombinant APOH led to a reduction in the proportion of CD86 cells and the protein level of iNOS (Fig. 7a–l). These findings suggest that pretreatment with APOH can partially inhibit M1 polarization in macrophages.

### APOH inhibits the TLR4/NF-κB pathway in macrophage cells treats with LPS

**Fig. 8:**
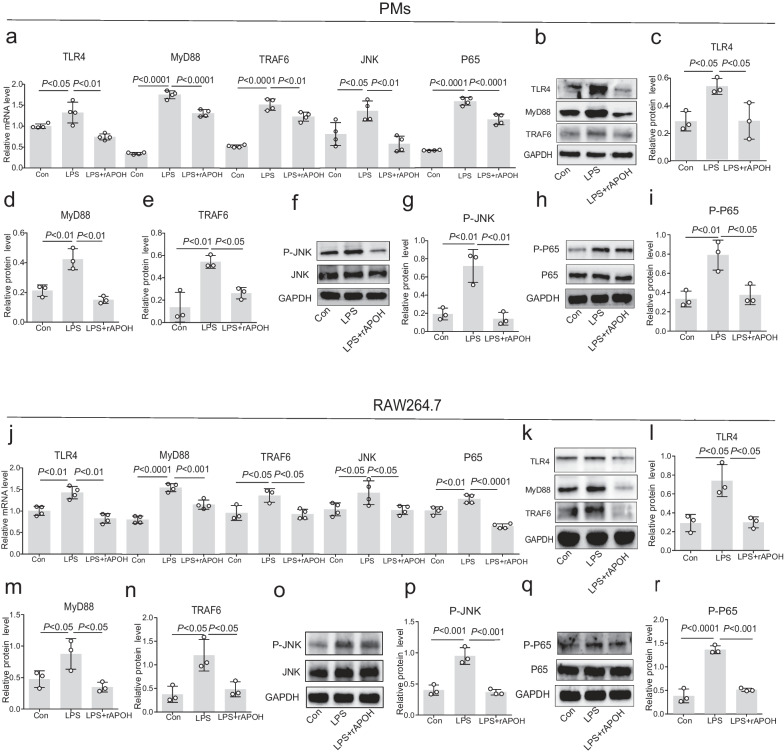
APOH inhibits the TLR4/NF-κB pathway in macrophage cells treats with LPS. **a** The mRNA expression levels of TLR4, MyD88, TRAF6, JNK and P65 in PMs. **b**–**e** Western blotting analysis of the relative expression of the TLR4, MyD88, and TRAF6 proteins in PMs. **f**–**g** Western blot analysis of the expression of phosphorylated JNK in PMs. **h**–**i** Western blot analysis of the phosphorylated P65 in PMs. **j** The mRNA expression of TLR4, MyD88, TRAF6, JNK and P65 in RAW 264.7 cells. **k**–**n** Western blotting analysis of the relative expression of TLR4, MyD88, and TRAF6 proteins in RAW 264.7 cells. **o**–**p** Western blot analysis of the expression of phosphorylated JNK in RAW 264.7 cells. **q**–**r** Western blot analysis of the expression of phosphorylated p65 in RAW 264.7 cells. Primer sequences in mRNA were listed in Additional file 5: Table S2. The data are presented as the means ± standard deviations (S.D.). “*” indicates a difference between groups. *p < 0.05, **p < 0.01, *** *p* < 0.001, **** *p* < 0.0001. Apolipoprotein H, APOH; peritoneal macrophages, PMs; lipopolysaccharide, LPS; Toll-like receptor 4, TLR4; myeloid differentiation factor 88, MyD88; nuclear factor-κB, NF-κB; TNF receptor associated factor 6, TRAF6; Jun N-terminal kinase, JNK

As a pattern recognition receptor, TLR4 plays a crucial role in the inflammatory response mediated by macrophages [13, 14]. Our results demonstrated an elevation in the mRNA levels of the TLR4/NF-κB pathway in PMs and RAW 264.7 cells after LPS stimulation. However, this effect was effectively mitigated by APOH (Fig. 8a and j). Furthermore, APOH pretreatment inhibited the protein levels of the TLR4/NF-κB pathway in PMs (Fig. 8b–i) and RAW 264.7 cells (Fig. 8k–r). These findings suggest the capacity of APOH to inhibit the TLR4/NF-κB pathway in macrophage cells treated with LPS.

## Discussion

In this study, we investigated the involvement of APOH in sepsis. We specifically focused on its impact on paediatric patients, an area that has not yet been previously explored. Our findings demonstrated that circulating levels of APOH decreased in paediatric patients with sepsis compared to healthy controls, a trend consistently observed in both the pilot and validation cohorts. Moreover, we observed that APOH levels remained low throughout the disease progression in the validation cohort. These results suggest that APOH is closely linked with paediatric patients with sepsis. Additionally, our investigation into the therapeutic potential of APOH administration in septic mice showed improved survival rates by reducing the expression of inflammatory cytokines (TNF-α, IL-1β, and IL-6) and mitigating tissue injuries. Conversely, neutralizing APOH activity with an anti-APOH antibody exacerbated the mortality rate in a non-severe sepsis model. Furthermore, our in vitro experiments demonstrated that APOH pretreatment regulated macrophage polarization by inhibiting the TLR4/NF-κB pathway upon LPS challenge without enhancing bacterial phagocytosis and killing capacities.

In previous studies, it has been suggested that APOH acts as an immunoregulatory factor by binding to the surfaces of apoptotic cells and bacteria [15]. Upon interaction with LPS, monocytes/macrophages phagocytose the APOH complex, resulting in a decrease in APOH levels [9, 10]. In this study, we observed a decrease in the relative abundance of APOH in patients with sepsis compared to healthy controls. Furthermore, we observed that patients with lower APOH levels exhibited higher mortality rates in a pilot cohort. In a study by Adolfo et al., an analysis of plasma proteins in sepsis patients and their correlation with organ dysfunction and mortality revealed that the decrease in APOH had a strong association with lower mortality. This suggested that APOH acts as a lipoprotein with a protective effect on mortality [16]. Similarly, El-Assaad et al. observed a decrease in APOH levels in male sepsis patients compared to healthy male controls. They further investigated this phenomenon using a murine model challenged with *Escherichia coli* (*E. coli*). They proposed that the reduced APOH level in males was due to APOH being functionally utilized as part of the immune system's protective response against *E. coli* [10]. These findings align with our observations.

The present study found significantly lower APOH levels in paediatric sepsis patients than in healthy controls, in the pilot and validation cohorts. Furthermore, the concentration of APOH at 48 and 72 h after admission was significantly decreased compared to that at 24 h in the validation cohort. However, there was no significant difference in APOH levels between the survivors and non-survivors. Interestingly, we observed distinct results in APOH levels between survivors and non-survivors in the pilot and validation cohorts. The differences between the two cohorts may stem from the inclusion of a smaller number of non-survivors in the validation cohort (n = 6) compared to the pilot cohort (n = 26 pooled to N = 6), potentially introducing bias into the APOH levels in the validation cohort. Additionally, Schrijver et al. demonstrated that APOH levels were decreased in patients with sepsis, particularly those with septic shock, without observing a significant difference between survivors and non-survivors [17]. Overall, these findings suggest a potential role of APOH in modulating the pathophysiology of sepsis, and continuous monitoring of APOH levels could aid in stratifying sepsis patients.

To further investigate the role of APOH in the pathogenesis of sepsis, we established a clinically relevant murine model of sepsis induced by CLP. Subsequently, we observed a decrease in APOH levels during the onset and progression of sepsis. However, the differences in APOH levels among the 24/48/72 h time points did not reach statistical significance. Several plausible reasons might underlie this discrepancy between paediatric sepsis patients and the murine sepsis model. Firstly, the decrease in APOH may be determined at earlier time points, such as 6, 12, or 18 h after CLP operation, considering the APOH content at 24 h had already sharply decreased to an extremely low level. Consequently, a further decrease may be challenging to observe at 48 or 72 h after CLP operation. Secondly, the higher number of included patients compared to the number of included mice could have influenced the statistical efficiency. Furthermore, our study revealed that mice subjected to CLP exhibited decreased expression of APOH in the lung, liver, and kidney compared to sham mice.

Our study demonstrated that supplementing with recombinant APOH increased survival rates and mitigated tissue inflammation and injuries in mice subjected to a severe CLP-induced sepsis model. Conversely, neutralizing APOH led to a higher mortality rate and increased systemic inflammation and tissue injuries in a non-severe CLP-induced sepsis model. Previous research has shown that APOH-deficient mice (*APOH*^−/−^) developed earlier onset of severe septicaemia than WT mice (male *APOH*^−/−^: 71.4% vs. male WT: 28.6%, with a severity score of ≥ 3) within 24 h following an intravenous injection of *E. coli* [10]. In a similar study, *APOH*^−/−^ mice displayed significantly higher levels of inflammatory cytokines than WT mice after an LPS challenge, and the total APOH levels were markedly decreased in WT mice after LPS injection compared to the saline control mice [18]. These findings strongly underscored that APOH is crucial in controlling bacterial infection in CLP-induced polymicrobial sepsis. This suggests that targeting APOH could be a promising novel therapeutic approach for sepsis treatment.

To elucidate the molecular mechanisms underlying the protective effect of APOH, we examined its impact on bacterial CFU levels and the abundance of infiltrating leukocyte types in septic mice. Our results showed no significant difference in bacterial CFU levels, but there was a tendency for an increase in macrophage abundance following APOH intervention. Macrophages, and other phagocytic cells are crucial in initiating the inflammatory process and clearing invasive bacteria [19, 20]. However, our study did not observe any direct effects of APOH on the phagocytosis and intracellular killing of live *P. aeruginosa* by macrophage. Previous studies have reported that APOH cannot kill bacteria directly, but peptides from domain V have exhibited antibacterial activities against *Streptococcus pyogenes* [21]. Conversely, other researchers demonstrated that APOH can enhance phagocytosis by binding to phosphatidylserine (PS)-containing vesicles or apoptotic cells, thereby promoting phagocytic engulfment [22]. These discrepancies suggest that the multiple functions of APOH may be mediated by its interaction with different partners in a cell-specific or disease-specific manner.

Macrophages undergo polarization into the M1 phenotype during the early stage of infection, leading to the release of inflammatory cytokines. However, to counteract the excessive inflammatory response, macrophages shift to the M2 phenotype to facilitate wound healing [23]. In our study, the supplementation of APOH decreased proinflammatory mediators TNF-α and IL-1β in various types of macrophages, including PMs, BMDMs, PBMCs, and RAW 264.7 cells. This prompted further investigation of the effect of APOH on macrophage polarization. Interestingly, we found that preincubation with recombinant APOH reduced M1 polarization without significantly increasing M2 polarization. The TLR4 signalling pathway is pivotal in releasing of inflammatory mediators such as IL-1, IL-6, TNF-α, and iNOS by activating the NF-κB signalling pathway in macrophages [24, 25]. Consequently, we explored whether the TLR4/NF-κB signalling pathway was involved in APOH-mediated macrophage polarization. Our results showed that APOH decreased the LPS-induced increase in the TLR4/NF-κB pathway. Overall, these findings suggest that APOH plays a protective role in sepsis by suppressing M1 polarization, at least partially, by inhibiting of the TLR4/NF-κB signalling pathway.

The present study has several limitations that merit consideration. Firstly, it was a single-center study with a relatively small sample size of sepsis patients. The absence of a significant difference in the APOH level between survivors and non-survivors underscores the necessity for a larger-scale clinical trial and further studies to investigate the potential discriminative role of APOH in sepsis patients. Secondly, for a more comprehensive understanding of the molecular mechanisms underlying local and systemic inflammation amplification during sepsis, additional research using APOH knockout mice is necessary. Lastly, it is essential to note that the ages of the healthy control group in the validation cohort were significantly higher than those in the sepsis group. This discrepancy was due to the difficulty in obtaining samples from healthy children under 3 years of age. Intriguingly, our study did not find a significant association between APOH levels and age in either sepsis patients or control subjects.

In summary, our study highlights a consistent and sustained reduction in serum APOH levels among paediatric sepsis patients, a phenomenon tightly linked to disease severity and mortality. Notably, the therapeutic administration of recombinant APOH demonstrated beneficial effects in a septic mouse model by reshaping macrophage polarization and inhibiting the intracellular activation of the TLR4/NF-κB signalling pathway. These findings provide valuable insights into APOH-driven regulation of the anti-inflammatory functions of macrophages, which might offer a novel therapeutic strategy for treating sepsis.

### Supplementary Information

**Additional file 1**. Detailed methods materials.

**Additional file 2**. **Table S1**: Patient characteristics in the pilot cohort.

**Additional file 3**. **Figure S1**: The levels of APOH and inflammatory cytokines in CLP mice. (a) Serum levels of IL-6 in Sham and CLP mice (n=5). (b) The correlation between APOH and IL-6. (c) Western blot analysis of the expression of APOH protein in the lungs, livers, and kidneys in CLP mice. The data were presented as the means ± standard deviations (S.D.). “*” indicated the difference between groups. *p < 0.05, **p < 0.01, *** p <0.001, **** p <0.0001. Apolipoprotein H, APOH; CLP, cecum ligation and puncture; IL, interleukin

**Additional file 4**. **Figure S2**: The abundance of infiltrating leukocytes and bacterial CFUs after the administration of recombinant murine APOH. (a) Flow cytometry was performed to determine the differences in the abundance of infiltrating leukocytes within the PLF in the BSA and rAPOH groups. (b) Bacterial CFUs of PMs and RAW 264.7 macrophages were treated with or without rAPOH and infected with P.a. Apolipoprotein H, APOH; recombinant APOH, rAPOH; CFU, Colony-Forming Units; Peritoneal macrophages, PMs; Peritoneal Lavage Fluids, PLF; Peripheral blood mononuclear cell; Pseudomonas aeruginosa, P.a

**Additional file 5.Table S2:** Primer sequences.

## Data Availability

The datasets used and/or analysed during the current study are available from the corresponding author upon reasonable request.

## Abbreviations

APOH: Apolipoprotein H
CLP: Cecal ligation and puncture
TLR4: Toll-like receptor 4
NF-κB: Nuclear factor-κB
WHO: World Health Organization
β2GPI: Beta-2-glycoprotein I
APS: Antiphospholipid syndrome
PICU: Paediatric intensive care unit
WBC: White blood cells
PCT: Procalcitonin
CRP: C-reaction protein
pSOFA: Paediatric sequential organ failure
PCIS: Pediatric critical illness score
SIRS: Systemic inflammatory response syndrome
BSA: Bovine serum albumin
rAPOH: Recombinant murine apolipoprotein H
PBS: Phosphate buffer solution
TNF-α: Tumour necrosis factor-alpha
IL-1β: Interleukin (IL)-1β
PLF: Peritoneal lavage fluids
H&E: Haematoxylin and eosin
CFU: Colony-forming unit
PMs: Peritoneal macrophages
RBCs: Red blood cells
BMDMs: Bone marrow-derived macrophages
GM-CSF: Granulocyte macrophage colony-stimulating factor
PBMCs: Peripheral blood mononuclear cells
FBS: Fetal bovine serum
LPS: Lipopolysaccharide
*P.a*: *Pseudomonas aeruginosa*
DAPI: 4′,6-Diamidino-2-phenylindole
TRAF6: TNF receptor associated factor 6
JNK: Jun N-terminal kinase
iNOS: Inducible nitric oxide synthase
Arg-1: Arginase
WT: Wild type
*E. coli*: *Escherichia coli*
PS: Phosphatidylserine
