# Supplementary material for: The protective effect of apolipoprotein H in paediatric sepsis
Source: Crit Care. 2024 Jan 30;28:36. doi: 10.1186/s13054-024-04809-2 (PMC10826270; doi:10.1186/s13054-024-04809-2)
Supplement: Supplementary file 1 — Additional file 1. Detailed methods materials. [file 13054_2024_4809_MOESM1_ESM.docx]

**Supplemental material**

**Methods**

**Enzyme-linked immunosorbent assay (ELISA)**

The human concentrations of APOH were quantified using commercial ELISA kits (Ruixin Biotech, RX103075, China). The levels of inflammatory cytokines in mice (n=7 per group), including tumour necrosis factor-alpha (TNF-α) (Neobioscience, EMC102a.96, China), interleukin (IL)-1β (Neobioscience, EMC001b.96, China), IL-6 (Neobioscience, EMC004.96, China) and IL-10 (Neobioscience, EMC005.96, China), in serum and peritoneal lavage fluid (PLF) were detected using commercially available ELISA kits.

**Histopathology**

Lung, liver, and kidney tissues were harvested, and haematoxylin and eosin (H&E) was used to assess pathological changes (n=7). Fresh samples were washed with cold PBS and fixed in 4% paraformaldehyde. Then, the tissues were dehydrated, embedded in paraffin, cut into 4 µm thick slices and stained routinely. The pathology scores of the lungs, livers, and kidneys were determined according to the following categories: lung histological alterations, including oedema, congestion, interstitial inflammation, and inflammatory cell infiltration, and liver and kidney histological alterations, including the numbers of thrombi and abscesses, inflammation, and necrosis (13, 14).

**Isolation of peritoneal macrophages, bone marrow-derived macrophages and peripheral blood mononuclear cells**

Mice were intraperitoneally injected with thioglycollate fluid culture medium to recruit macrophages. For isolation of peritoneal macrophages (PMs), peritoneal lavage was performed with 5 ml of PBS. The peritoneal lavage fluid (PLF) was centrifuged at 500 ×g for 10 min at 4 °C, and then, the erythrocytes were lysed with red blood cell (RBC) lysis buffer (Tiangen, RT122-02, China) for 5 minutes at room temperature. The sediments were obtained at 500 ×g for 10 minutes at 4 °C and washed with PBS. Then, the cells were harvested by plastic adherence.

Mouse bone marrow-derived macrophages (BMDMs) were isolated from the femur and tibia of C57BL/6 mice and differentiated into macrophages by 50 ng/ml recombinant granulocyte macrophage colony-stimulating factor (GM-CSF) (MedChemExpress, HY-P7361, USA) in DMEM (Gibco, Life Technologies, Germany).

Peripheral blood mononuclear cells (PBMCs) were isolated from blood buffy using a Lympholyte®-H (Cedarlane, CL5020-R, Canada) through a density gradient. The PBMCs were washed twice with 1640 (Gibco, Life Technologies, Germany) containing 2% foetal bovine serum (FBS) and incubated in erythrocyte lysis buffer for 5 minutes at room temperature. Subsequently, PBMCs were suspended in RPMI 1640 medium containing 10% FBS and differentiated into macrophages with 20 ng/ml recombinant GM-CSF.

**Phagocytosis function assay**

PMs, PBMCs and RAW 264.7 cells were seeded in 24-well plates and incubated with rAPOH for 20 hours. Cells were infected with fluorescein isothiocyanate–labelled *P. aeruginosa* (P.a) at a multiplicity of infection (MOI) of 100 for 30 minutes at 37 °C and washed and stained with 4′,6-diamidino-2-phenylindole (DAPI) (Beyotime, China, C1005) and TRITC Phalloidin (Solarbio China, CA1610), followed by visualization using confocal laser scanning microscopy (Nikon C2+).

**Bacterial killing assays**

For determination of bacterial killing by macrophages, PMs and RAW 264.7 macrophages (1x105 cells) were preincubated with recombinant mouse APOH for 20 hours and infected with *P. aeruginosa* (P. a) (MOI, 1:100) in a 37 °C incubator for 30 minutes at 70 revolutions per minute (RPM), and then, the cells were washed with buffer containing gentamycin (200 µg/ml) for 15 minutes at 4 °C to remove extracellular bacteria and washed 3 times in PBS. Then, the cells were lysed with sterile double distilled water for 20 minutes at room temperature. Live intracellular bacteria were calculated to quantitate bacterial killing by culture of lysates at 37 °C for 2 hours. Lysates were cultured on blood-agar plates overnight at 37 °C, and CFU counts were counted. The killing rate= (number of CFUs=0 hour-number of CFUs=2 hours)/number CFUs=0 hour was calculated.

**RNA extraction and quantitative real-time PCR**

Total RNA was extracted from cells using the RNA Isolation Kit (Beyotime, R0027, China) and reverse transcribed to cDNA by using ABScript III RT Master Mix for qPCR with a gDNA remover kit (ABclonal, RK20429, China) from 1 μg RNA. Real-time quantitative PCR (qPCR) was performed by using SYBR Green (ABclonal, Rk21203, China) to detect gene expression on a Bio-Rad CFX ConnectTM Real-Time System (Bio-Rad, USA). The primer sequences are listed in Additional file 2: Supplemental Table 2, and the results were calculated by the 2−△△Cq method with normalization to GAPDH.

**Bacterial colony-forming units**

Dilutions of peripheral blood, PLF, liver, lung, or kidney of mice were plated on blood-agar plates overnight at 37 °C. Colony-forming unit (CFU) counts were then determined after 24 hours of culture (n=7 per group) (13).

**Flow cytometry experiments**

We investigated whether any observable differences in the abundance of infiltrating leukocytes after CLP within the PLF between the rAPOH and BSA groups could be observed (n=7 per group). Peritoneal macrophages were collected and incubated with monoclonal antibodies against CD16/32 (Biolegend, 101302, USA), CD11b (Biolegend, 101212, USA), isotypic CD11b (Biolegend, 400611, USA), Ly6G (Biolegend, USA, 127605), isotypic Ly6G (Biolegend, 400505, USA), F4/80 (Biolegend, 123110, USA) and isotypic F4/80 (Biolegend, 400507, USA).

In the in vitro experiment, RAW 264.7 and PMs cells were preincubated with rAPOH for 30 minutes and treated with LPS for 6 hours. Then, the cells were adjusted to 106/μl, and 100 μl specimens and 1 μl of F4/80, CD11b, CD86 (Biolegend,105013, USA), and CD206 (Biolegend, 141709, USA) antibodies were added and incubated at room temperature for 30 minutes in the dark and examined by flow cytometry (FACSVerse™, BD Biosciences).

**Western blotting analysis**

Proteins were harvested from cells by using radioimmunoprecipitation assay (RIPA) lysis buffer (MCE, HY-K1001, USA) with 1% phenylmethanesulfonylfluoride (PMSF) (Beyotime, ST506, China), 1% protease inhibitor (MCE, HY-K0010, USA) and 1% phosphatase inhibitors (MCE, HY-K0021, USA). The protein concentration was measured with a NanoDrop spectrophotometer (Thermo Fisher). The proteins were separated by sodium dodecyl sulphate‒polyacrylamide (SDS‒PAGE) gel (EpiZyme Biotechnology, PG112, China) electrophoresis and then transferred onto a polyvinylidene fluoride (PVDF) membrane (Millipore, IPVH00010, USA). The membranes were blocked in NcmBlot blocking buffer (New Cell & Molecular Biotech, P30500, China) for 20 minutes and incubated with corresponding primary antibodies overnight at 4 °C. The following primary antibodies were used: anti-TLR4 (Proteintech, 66350-1-Ig, China), anti-TNF receptor associated factor 6 (TRAF6) (Zen-Bio, 380803, China), anti-Myd88 (Zen-Bio, 340629, China), anti-Jun N-terminal kinase (JNK) (Abcam, USA, ab124956), anti-phospho-c Jun N-terminal kinase (p-JNK) (Abcam, ab179461, USA), anti-nuclear factor kappa-B (NF-κB) p65 (Zen-Bio, R25149, China), anti-phospho-p65 (Cell Signaling Technology, 3033T, USA), anti-IL-1β (Proteintech, 26048-1-AP, China), anti-TNF-α (Proteintech, 17590-1-AP, China), anti-inducible nitric oxide synthase (iNOS) (Zen-Bio, 340688, China), and anti-arginase (Arg-1) (Zen-Bio, R380744, China). Then, the membranes were incubated with goat anti-rabbit HRP-conjugated polyclonal antibody (Hua Bio, ET1601-4, China) and goat anti-mouse HRP-conjugated polyclonal antibody (Proteintech, 66009-1-Ig, China) secondary antibodies at room temperature for 1 hour separately and visualized by a Bio-Rad ChemiDocTM Touch Imaging System (Bio-Rad, California, USA).
