# Supplementary material for: The protective effect of apolipoprotein H in paediatric sepsis
Source: Crit Care. 2024 Jan 30;28:36. doi: 10.1186/s13054-024-04809-2 (PMC10826270; doi:10.1186/s13054-024-04809-2)
Supplement: Supplementary file 2 — Additional file 2. Table S1: Patient characteristics in the pilot cohort. [file 13054_2024_4809_MOESM2_ESM.docx]

**Supplemental Table1** Characteristics of patients with sepsis and healthy controls in pilot cohort

| Characteristics | Sepsis survivors | Sepsis nonsurvivors | Healthy control | *P* value |
| --- | --- | --- | --- | --- |
| Number (n) | 42 | 26 | 26 |  |
| Sex (male/female) | 25:17 | 15:11 | 16:10 | n.s |
| Age (years) | 2.54 (0.48-11.25) | 3.1(0.7-9.6) | 2.4(1.6-4.9) | n.s |
| lactate (mmol/L) | 1.8 (1.3-4.1) | 5 (2.3-7.5) | N/A | 0.003 |
| PCT (ng/ml) |  |  | N/A |  |
| ＞100（n, %） | 18 (42.8%) | 11 (42%) |  |  |
| <100 | 14.72 (0.55-25.84) | 11.12 (0.9-28.4) |  |  |
| potential diseases | 12 (28.5%) | 14 (54%) | N/A |  |
| Organs dysfunction（n, %） |  |  | N/A |  |
| Respiratory | 9 (80.9%) | 26 (100%) |  |  |
| Circulatory | 32 (76.1%) | 23 (88%) |  |  |
| Nervous | 9 (21.4%) | 16 (62%) |  |  |
| Blood | 30 (71.4%) | 24 (92%) |  |  |
| Gastrointestinal | 10 (23.8%) | 18 (69%) |  |  |
| Urinary | 29 (69%) | 21 (81%) |  |  |
| Bacteremia |  |  | N/A |  |
| Gram positive | 11 (26.1%) | 8 (31%) |  |  |
| Gram negative | 13 (30.9 %) | 8 (31%) |  |  |
| Fungus | 2 (4.76) | 2 (8%) |  |  |
| Bloodculture（n, %） | 10 (23.8%) | 12 (46%) | N/A |  |
| Infection site  (n, %) |  |  | N/A |  |
| Respiratory | 15 (35.7%) | 11 (42%) |  |  |
| Gastrointestinal | 14 (33.3%) | 6 (23%) |  |  |
| Nervous | 2 (4.76%) | 6 (23%) |  |  |
| Blood | 5 (11.9%) | 1 (4%) |  |  |
| Skin | 5 (11.9%) | 2 (8%) |  |  |
| Vascular | 1 (2.38%) | 0 |  |  |
| ICU stay(days) | 11.5(6.0-17.0) | 3(0.92-8.25) | N/A |  |

NOTE: Data are expressed as median (interquartile range) unless otherwise indicated. PCT: procalcitonin; ICU: intensive care unit; n.s: No significant; NA: not applicable.
