# Supplementary figures and images for: The protective effect of apolipoprotein H in paediatric sepsis
Source: Crit Care. 2024 Jan 30;28:36. doi: 10.1186/s13054-024-04809-2 (PMC10826270; doi:10.1186/s13054-024-04809-2)

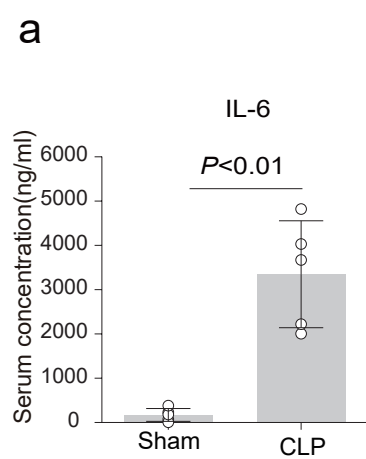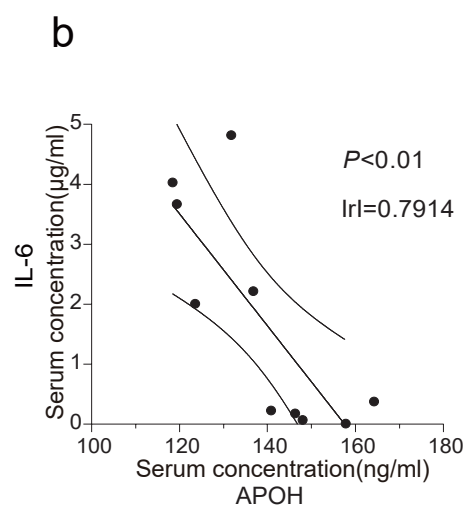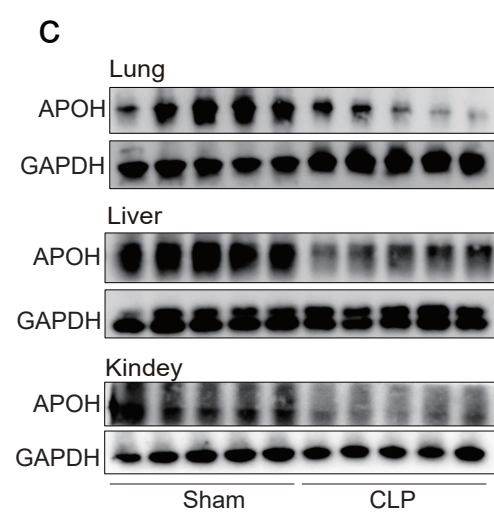

Supplement: Supplementary file 3 — Additional file 3. Figure S1: The levels of APOH and inflammatory cytokines in CLP mice. (a) Serum levels of IL-6 in Sham and CLP mice (n=5). (b) The correlation between APOH and IL-6. (c) Western blot analysis of the expression of APOH protein in the lungs, livers, and kidneys in CLP mice. The data were presented as the means ± standard deviations (S.D.). “*” indicated the difference between groups. *p < 0.05, **p < 0.01, *** p <0.001, **** p <0.0001. Apolipoprotein H, APOH; CLP, cecum ligation and puncture; IL, interleukin [file 13054_2024_4809_MOESM3_ESM.pdf]

a

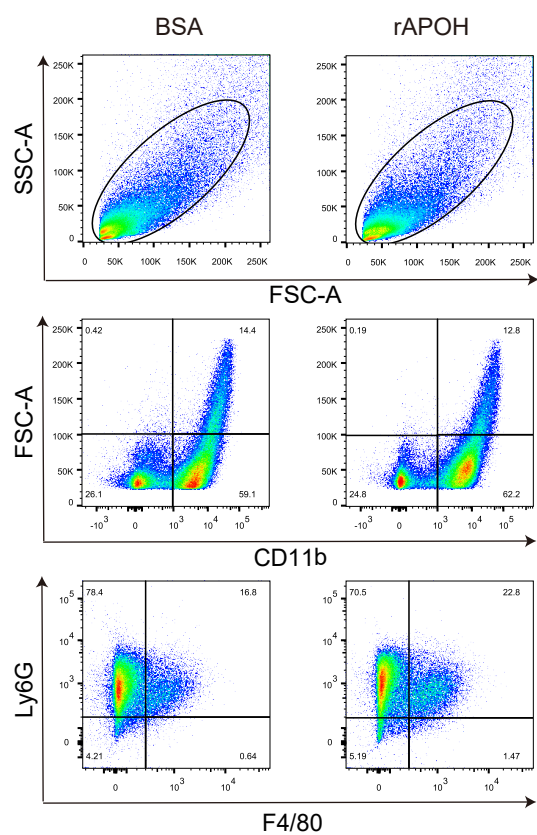

b

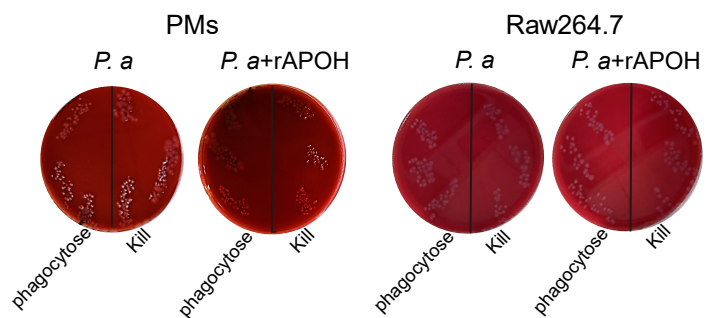

Supplement: Supplementary file 4 — Additional file 4. Figure S2: The abundance of infiltrating leukocytes and bacterial CFUs after the administration of recombinant murine APOH. (a) Flow cytometry was performed to determine the differences in the abundance of infiltrating leukocytes within the PLF in the BSA and rAPOH groups. (b) Bacterial CFUs of PMs and RAW 264.7 macrophages were treated with or without rAPOH and infected with P.a. Apolipoprotein H, APOH; recombinant APOH, rAPOH; CFU, Colony-Forming Units; Peritoneal macrophages, PMs; Peritoneal Lavage Fluids, PLF; Peripheral blood mononuclear cell; Pseudomonas aeruginosa, P.a [file 13054_2024_4809_MOESM4_ESM.pdf]
