# Supplementary material for: The protective effect of apolipoprotein H in paediatric sepsis
Source: Crit Care. 2024 Jan 30;28:36. doi: 10.1186/s13054-024-04809-2 (PMC10826270; doi:10.1186/s13054-024-04809-2)
Supplement: Supplementary file 5 — Additional file 5.Table S2: Primer sequences. [file 13054_2024_4809_MOESM5_ESM.docx]

| Gene | Forward primer | Reverse primer |
| --- | --- | --- |
| GAPDH | ACCCAGAAGACTGTGGATGG | CACATTGGGGGTAGGAACAC |
| TNFα | ATGTCTCAGCCTCTTCTCATTC | GCTTGTCACTCGAATTTTGAGA |
| IL-6 | CTCCCAACAGACCTGTCTATAC | CCATTGCACAACTCTTTTCTCA |
| IL-1β | CACTACAGGCTCCGAGATGAAC | TGTCGTTGCTTGGTTCTCCTTGT |
| TLR4 | CACAGAAGAGGCAAGGCGACAG | GAATGACCCTGACTGGCACTAACC |
| MyD88 | AGCAGAACCAGGAGTCCGAGAAG | GGGCAGTAGCAGATAAAGGCATCG |
| TRAF6 | ACAGCAACTCTTACAGCCAGGAAAC | AACCACTGAGCCAATTCTCCAACC |
| JNK | CACAGTGAGCAGAGCAGGCATAG | TTGTCAGGAGCAGCACCATTCTTAC |
| NF-κB/ P65 | AGACCCAGGAGTGTTCACAGACC | GTCACCAGGCGAGTTATAGCTTCAG |

**Supplemental Table 2.** **Primers sequences for the real-time PCR**

NOTE: GADPH: glyceraldehyde-3-phosphate dehydrogenase, IL-1β: interleukin-1β, IL-6: interleukin-6, TNF-α: tumor necrosis factor alpha, TLR4: toll-like receptor 4, MyD88: myeloid differentiation factor 88, TRAF6: TNF receptor associated factor 6, JNK: Jun N-terminal Kinase, NF-κB/P65: nuclear factor kappa-B p65
